# A Study of Enamel Defects and Dental Caries of Permanent Dentition in School Children with Intellectual Disability

**DOI:** 10.3390/jcm9041031

**Published:** 2020-04-06

**Authors:** Karolina Gerreth, Justyna Opydo-Szymaczek, Maria Borysewicz-Lewicka

**Affiliations:** Department of Risk Group Dentistry, Chair of Pediatric Dentistry, Poznan University of Medical Sciences, 70 Bukowska Street, 60-812 Poznan, Poland; jopydo@ump.edu.pl (J.O.-S.); klstomdz@ump.edu.pl (M.B.-L.)

**Keywords:** developmental enamel defects, dental caries, children, intellectual disability, special-care schools

## Abstract

Data concerning the prevalence of developmental enamel defects and their association with dental caries in individuals with intellectual disability are scarce. This paper aims to evaluate the prevalence and distribution of developmental enamel defects and dental caries in the permanent dentition of special-care school children from Poznan (Poland). Out of 1091 students attending all special-care schools in the city, the study covered 268 subjects with intellectual disability (mild, moderate, severe, and profound) with permanent dentition, aged 10–20. One calibrated dentist performed dental examinations. The Statistica Software v10 was used for statistical analysis, assuming the level of statistical significance *p* ≤ 0.05. Among the subjects of the study, 19.40% presented developmental enamel defects. The number of teeth with changes ranged from 1 to 28, with maxillary incisors most frequently affected. Students without developmental enamel defects had more teeth observed with active caries compared to those with such changes (10.92% vs. 7.82%, *p* < 0.01). The highest number of students with developmental defects of enamel was observed in the group of individuals with mild intellectual disabilities. The present study revealed that in special-care students from Poznan, enamel defects and dental caries were frequently observed. However, individuals with developmental enamel defects did not show higher dental caries indices.

## 1. Introduction

The developing dental enamel is very susceptible to different systemic and local factors, and is unable to regenerate after damage [1,2,3]. Since odontogenesis begins in utero and ends around the age of 18–25 years, defects of enamel may result from a wide variety of insults that affect the tooth from before the birth to adulthood [1]. The lesions may be localized or generalized, qualitative or quantitative, depending on a type of insult and the stage of amelogenesis. Causative agents of localized defects include traumatic injuries, local infections, and irradiation. Defects with the generalized type of distribution may be caused by genetic disorders or by environmental intoxicants such as fluoride and dioxins as well as systemic disturbances, including perinatal and postnatal problems, malnutrition, infectious diseases, and a range of other medical conditions [1,4,5,6,7].

Several systemic factors that disrupt neurological development may also alter amelogenesis [2]. Enamel defects associated with various degrees of intellectual disability are typical of several genetically determined diseases, such as velocardiofacial syndrome (22q11.2 deletion syndrome), the Kenny Caffey syndrome, as well as Kohlschütter-Tönz syndrome [8,9]. The risk of acquired enamel defects has also been discussed in the context of brain development. Already half a century ago, Cohen and Diner noticed that enamel defects occurred with higher frequency in children with IQ deficits compared to neurologically healthy children [10]. Their study and further reports suggested that affected enamel may give information concerning the timing of insults, possibly influencing other structures of ectodermal origin, such as the brain [2,3,11,12]. The ameloblasts might respond to different factors in a similar way, and the exact nature of the insult often remains unknown.

However, there are speculations in the literature that the chronologically distributed developmental defects of enamel (DDE) might be a significant aid in the neurological diagnosis, especially since many cases of intellectual disability are of unknown etiology [10,11,12,13]. Therefore, dental examination might help to establish the time of the possible harmful health event contributing to mental retardation, although it gives little information on the event itself.

Research studies confirmed that the presence of enamel hypoplasia and molar incisor hypomineralization increase the risk of dental caries [14,15]. The anomalous structure and morphology of teeth, caused by incorrectness during the process of amelogenesis, might contribute to the initiation and progression of caries [14,15]. Regarding diffuse fluoride-opacities and genetically determined amelogenesis imperfecta, some studies revealed that they are accompanied by low dental indices [16,17,18].

Data concerning the prevalence of enamel defects in the population of children and adolescents with intellectual disability are sparse [2,3,11,12,19], with no data concerning the association of developmental defects of enamel and dental caries. 

Therefore, the aim of the study was to evaluate the occurrence of DDE and dental caries in the population of subjects with intellectual disability attending special-care schools and residing in one large city (Poznan, Wielkopolska province, western Poland).

## 2. Material and Methods

### 2.1. Study Population

The study was carried out among the students with varying degrees of intellectual disability and coexisting other deficiencies and/or different systemic diseases, attending all eight special-care schools in the city of Poznan (Wielkopolska province, western Poland). There were a total of 1091 students in those institutions, aged between 6 and 25.

According to statistical data, at the time of the study, the total Wielkopolska province population comprised of 3,378,502 people. Among 564,951 citizens of Poznan, there were 130,347 individuals between the age of 5 and 24 [20]. 

The data concerning the degree of the individual’s intellectual disability (mild, moderate, severe, profound) among students were obtained from school’s records. Before the study, approval from the Ethical Committee of the Poznan University of Medical Sciences (resolution no. 783/06) was obtained, as well as consent from the heads of special-care schools and children’s parents or legal caregivers. Each child’s parent or legal caregiver was provided with data concerning the examination during the parent-teacher meeting and gave written and informed consent. Full confidentiality of the collected information was provided to the participants of the research. 

The researcher visited each special-care school a few times to carry out teeth evaluation since there were too many students at the institutions and some of them were absent during the day of examination. Therefore, there was a chance to examine students that were absent at the school during previous visits to the examiner.

Finally, out of the overall number of 1091 students attending special-care schools, 493 (45.19%) obtained written consent for dental examination from parents or legal caregivers. Teeth assessment was carried out in 379 individuals since other students were absent at school at the days of examination (19 subjects), or they were uncooperative (95 subjects). Therefore, their dentition could not be assessed. Mostly, subjects over the age of 18 years old were not cooperative since they were severely or profoundly intellectually disabled with multiple defects. 

Nonetheless, in the present study, we took into consideration 268 persons with permanent dentition (aged 10–20 years old), assuming that in the case of children with mixed dentition (95 subjects), the results may be underestimated since unerupted teeth might have some defects of enamel. Some students with permanent dentition had extensive carious changes (7 subjects) or numerous extracted teeth (9 subjects), and had to be excluded from the analysis. Some of the students lacked second molars.

All children and adolescents were of Caucasian origin, with no ethnic, cultural, demographic, or regional differences. Inclusion and exclusion criteria for the subjects in the study are presented in Table 1.

The inclusion criteria to the study consisted of informed and written consent of the child’s parent or legal caregiver, student’s consent, and expression of his/her willingness to participate in dental examination as well as the presence of permanent dentition. Excluded from the research were students that refused to participate or failed to cooperate or those that were absent from the institution during the days of examination (the dentist visited each special-care school several times). The exclusion criteria also included the presence of any deciduous tooth. 

The mean age of the total population of 268 students (118 females (44.03%) and 150 males (55.97%)) with permanent dentition who participated in the study was 14.77 (SD = 2.14; min = 10; max = 20), and in the group of 146 individuals (54.48%; 70 females and 76 males) with mild disability, it was 14.42 (SD = 1.88; min = 10; max = 18), in 84 students (31.34%; 33 females and 51 males) with moderate disability, it was 14.84 years (SD = 2.14; min = 10; max = 19), in 29 persons (10.82%; 9 females and 20 males) with severe disability, it was 16.31 years (SD = 2.62; min = 11; max = 20), and in 9 people (3.36%; 6 females and 3 males) with profound disability, it was 14.78 years (SD = 2.54; min = 12; max = 18).

Caregivers were asked to complete a questionnaire concerning the general disorder of the child, other coexisting diseases, as well as the drugs used. However, due to many missing answers, we were not able to use the collected data in order to assess the correlation between enamel defects and other health problems.

In Poznan, tap water is not artificially fluoridated, and its quality is constantly supervised by the State Sanitary Inspector [21]. In recent years, fluoride level in drinking water in the city has oscillated between 0.1 and 1.0 ppm; in 1996, it was 0.1–0.6 ppm, and in 1997 it was 0.2–1.0 ppm, whereas in the 2nd quarter of 2015 it was between 0.14 and 0.56 ppm [22,23].

The concentration of such pollutants as polychlorinated biphenyls, polybrominated diphenyl ethers, and organochlorine pesticides in human samples from the Wielkopolska region is lower in comparison with other European countries [7]. 

### 2.2. Degree of an Intellectual Disability Evaluation

An evaluation of a need for special education is made at a written request of child’s parents or caregivers with an attachment of any previous medical or psychological evaluations, and other relevant documentation [24]. The decision concerning such a necessity is issued after a psychological and pedagogical examination that is carried out by specialists from assessment boards operating in public psychological and educational counselling centers [24,25,26]. After such evaluation, a child receives a written statement recommending special form of education.

The degree of intellectual disability is evaluated, by the specialist in psychology, based on the ICD-10 classification of intellectual disability of the World Health Organization [24,27]. Under the decision of specialists from a psychological and educational counselling center, children are allocated to a specific category of students, i.e., particular classes.

### 2.3. Information from the Questionnaires Concerning General Diseases of Subjects

As a part of the study, the researchers distributed questionnaires to parents/caregivers concerning general and other coexisting diseases of the subjects. However, most of respondents did not return the forms or did not provide all necessary information. Therefore, it was impossible to include these data in results analysis. Thus, we presented them only hereunder. 

Out of 52 parents/caregivers of subjects with DDE, 8 (15.38%) respondents did not return the questionnaires at all, 10 (19.23%) persons did not give any information concerning the general disease or other accompanied disorders of the child, 19 (36.54%) parents/caregivers wrote that their children did not have any other disorder except intellectual disability; whereas 12 (23.08%) informed that children had cerebral palsy and/or epilepsy, and single individuals had autism, Marshall-Smith syndrome or cri du chat syndrome. 

In the case of parents/caregivers of 216 subjects without DDE, 39 (18.05%) respondents did not return the questionnaires, 40 (18.52%) individuals did not give any information concerning the general disease or other accompanied disorders of their child, 20 (9.26%) persons wrote that their children did not have any other disorder except intellectual disability, 54 (25.00%) had cerebral palsy and/or epilepsy, 5 (2.31%) had attention deficit hyperactivity disorder (ADHD), 9 (4.17%) had autism, 14 (6.48%) had Down syndrome, 2 (0.92%) subjects had Bourneville-Pringle syndrome, and 33 (15.29%) had multiple defects.

### 2.4. Clinical Examination

The clinical examination was carried out at a special-care school, in the nurse’s office. The participation of each student in the study was voluntary. The dental evaluation was carried out, using “tell, show, do technique”, and positive reinforcement, without any pharmacological preparation or physical restraints, and it was not performed if the child refused to participate or failed to cooperate. 

An intraoral examination was carried out by one dentist, as in other studies concerning chronically ill patients [28], over six months. The examiner (K.G.) underwent training and calibration concerning the diagnosis of developmental defects of enamel and dental caries by another experienced specialist in pediatric dentistry (M.B.L.) before the research. For this, firstly, M.B.L. carried out theoretical preparation to the research concerning the evaluation of caries lesions as well as developmental defects of enamel diagnosis according to WHO (World Health Organization) guidelines [29]. Secondly, K.G. examined the group of 25 generally healthy children at the Department of Pediatric Dentistry, Poznan University of Medical Sciences. The intra-examiner agreement concerning caries and defects of enamel was evaluated based on another dental check-up in the same group of 25 children after two weeks, with a κ of 0.95 and 0.97, respectively. Subsequently, K.G. carried out the dental examination at special-care schools. Additionally, to improve the intra-examiner agreement concerning examined lesions in a population of patients with disability, the group of 25 patients was examined by K.G. two weeks later, with a κ of 0.97 and 1.00, respectively.

The dental evaluation was performed in the artificial light of a headlamp, using a ball ended dental explorer and a plane mouth mirror. The data were recorded in a dental chart, specially designed for the study. 

The child was placed in a chair with his/her head resting against the wall, and in some cases, the teacher or nurse helped to stabilize the head. 

Teeth were inspected wet without previous professional cleaning. In some cases, the cotton roll was used to remove the debris. A tooth was evaluated as erupted when over half of the crown was present within the oral cavity [30]. 

The examination of enamel defects was carried out visually and had a character of screening test without recording separate categories of demarcated and diffused opacities and hypoplasia (examiner marked enamel defect in patient’s chart if any developmental abnormality of enamel was visible on any surface of a tooth). The prevalence of enamel defects was determined by the inclusion of any individual who has been found to have at least one tooth affected by the condition.

Developmental defects of enamel were easily distinguished from white spot lesions on clinical grounds, based on the association of the carious lesion located on a tooth and areas of mature plaque [4].

Individuals with enamel defects were divided into two subgroups. The first one included subjects with at least one defect of early developing teeth (i.e., first molars and incisors), while the second one included subjects with defects limited to later developing teeth (canines, premolars or second molars). In the case of the general involvement of early and later developing teeth, the subject was assigned to the first group.

The dental caries experience was assessed based on the number of decayed (DT), missing (MT), and filled teeth (FT) (DMFT index - a sum of DT, MT, and FT). If a tooth had temporary restoration, it was calculated as DT only. Caries was recorded as present when respective lesion showed an undermined enamel, unmistakable cavity, or a detectably softened wall or floor [29]. A probe was used to confirm visual evidence of caries. Areas with visual evidence of demineralization, presenting no soft surface, were considered sound. Tooth filled due to decay was recorded when a tooth had at least one permanent restoration placed to treat caries. The missing (MT) component was recorded when a tooth had been extracted due to caries complications (verified by interview).

The data obtained were used to calculate caries prevalence and severity, and prevalence and distribution of defects of enamel in particular teeth groups. 

After the examination, parents received standardized written information concerning oral health status and treatment needs of their children. Therefore, parents/legal caregivers of patients obtained the recommendation to visit the dentist for prophylactic and/or therapeutic procedures.

### 2.5. Statistical Analysis

Data from the research were coded and entered into an Excel spreadsheet, and subsequently double-checked to verify their accuracy. Statistical analysis, using the difference test between two proportions and the Statistica Software v 10 (StatSoft Inc., Tulsa, Oklahoma, USA), took into account the number of individuals with enamel defects in subgroups presenting various degrees of intellectual disability (i.e., mild, moderate, severe, and profound), the percentage of subjects with defects of early developing teeth and subjects with defects limited to later developing teeth, number of teeth with enamel defects, number of teeth groups affected by enamel lesions with respect to the dental arch (maxillary and mandibular) and to the side of the oral cavity (right and left) as well as differences between particular groups of teeth. Moreover, statistically significant differences concerning dental caries occurrence between individuals with developmental enamel defects and those free of such changes were calculated for the dental arch, side of the oral cavity as well as groups of teeth. A Mann–Whitney U test was used to test the differences between DMFT scores of students with mild and moderate to profound disability. A value of *p* ≤ 0.05 was considered statistically significant.

## 3. Results

Enamel defects were diagnosed in 52 individuals (19.40% of the total examined population), including 25 females and 27 males. The statistically significant difference was observed between the percentage of affected special-care students with mild (24.66%) and moderate (13.09%) or moderate to profound intellectual (13.11%) disability (*p* = 0.03 and *p* = 0.02, respectively) (Table 2).

Percentage of females and males with DDE did not differ statistically (21.20% vs. 18.00%, respectively, *p* > 0.05). The mean age of students with DDE and without DDE was similar (14.67 vs. 14.80, respectively, *p* > 0.05) (Table 3).

The percentage of students with moderate to profound disability who showed defective enamel of first permanent molars and incisors was statistically significantly higher as compared to the percentage of individuals with mild disability and defects of early developing teeth (93.75% vs. 60.53%, *p* = 0.02, respectively) (Table 4).

DMFT index of students with respect to the degree of intellectual disability was presented in Table 5. Mann–Whitney *U* test did not reveal statistically significant differences between the DMFT numbers of subjects with mild and more advanced degrees of intellectual disability (4.56 ± 3.85 vs. 5.09 ± 4.42, *p* > 0.05, respectively). 

The calculated DMFT index for students affected by developmental enamel defects was 3.86 ± 2.69 (mean ± SD), with DT = 2.17 ± 2.05, MT = 0.19 ± 0.66, and FT = 1.50 ± 2.21. In those without enamel defects, the index amounted to 5.03 ± 4.37, and the values of particular components were as follows: 2.96 ± 3.12, 0.58 ± 2.70, and 1.49 ± 2.24, respectively.

In the group of subjects with developmental enamel defects, only six (11.54%) students did not suffer from dental caries (DMFT = 0), including five individuals with mild and one with moderate intellectual disability. The other subjects with DDE (88.46%) had from one to 12 teeth with caries and/or extracted and/or filled due to the carious process. 

In total, students with defects of enamel had 1445 permanent teeth present in their oral cavity (723 maxillary teeth and 722 mandibular teeth; 723 teeth on the right and 722 on the left side of the oral cavity), and in particular individuals, the values were from 22 to 32 teeth (mean = 27.98; SD = 1.80). 

Subjects presented from one to 28 teeth (mean = 8.27; SD = 8.88) with defective enamel. In five (9.61%) students (two females and two males with mild intellectual disability and one female with moderate intellectual disability) all teeth present in the oral cavity were affected (one individual had 27 teeth with lesions, whereas the other four students 28 teeth).

The most frequently students had two teeth with enamel defects (21.15%), then one tooth (15.39%) and four teeth (13.46%) with such changes, and the statistical significance was observed between the number of individuals with two defects and the number of children with three and five to 27 defects (*p* < 0.05) (Table 6).

In the group of subjects with one tooth affected with DDE, the defects prevailed on upper incisors (in three persons in tooth 21 and in one student in tooth 11). Other subjects had defects of enamel in teeth 12, 23, 24 and 45. Among 11 individuals with two teeth affected, five students had symmetric lesions on both upper central incisors. In 41 students (78.85% of the total population with defects) with several teeth affected, the teeth from the same groups presented defects, i.e., incisors and/or canines and/or premolars and/or molars.

Enamel defects were seen less frequently in mandibular incisors (12.98%), while maxillary incisors were the most commonly affected (49.52%), and the difference was statistically significant (*p* < 0.001) (Table 7). 

In total, 430 teeth (29.76%), out of the 1445 present in students, had developmental defects of enamel. Defects in mineralization were more often seen in maxillary teeth than in mandibular ones (36.65% and 22.85%, respectively; *p* < 0.001) (Table 7).

There was the same number of teeth (215) in the right and left side of the oral cavity with enamel lesions (29.74% and 29.78%, respectively) (Table 7).

Out of 52 subjects with enamel defects, as many as 19 (36.54%) students had lesions limited to incisors, whereas in 19.23% of individuals the changes were seen in all teeth groups (i.e., incisors, canines, premolars, and molars) (Table 8).

In the total population affected by enamel defects, six (11.54%) students did not have any other pathological changes within the dentition, while 39 (75.00%) individuals suffered from dental caries. Nine (17.31%) children and adolescents had both enamel defects and caries in the same tooth (20 teeth, 4.65% out of 430 teeth with enamel defects). Five (9.61%) students with enamel defects had the teeth missing due to caries, and 19 (36.54%) individuals had teeth restored due to caries, including six (11.54%) children that had both enamel defect and filling in the same tooth (15 teeth, 3.49% out of 430 teeth with enamel defects). 

When considering the number of teeth with caries, fillings, and extracted due to decay in both arches, mandibular molars showed higher caries experience as compared to maxillary molars (*p* < 0.001) in both groups, i.e., with and without DDE (Table 9). In the group without enamel defects, maxillary incisors were more frequently affected by caries as compared to lower incisors (*p* < 0.001).

In the total population of students with DDE, 88,46% subjects were affected by dental caries, with DMFT amounting to 3.86 (DT = 2.17; MT = 0.19; FT = 1.50), whereas in subjects that were free of enamel defects, the values were 88.89% and 5.03 (2.96; 0.58; 1.49), respectively, *p* > 0.05. 

## 4. Discussion 

Since different factors may affect the development of tooth hard tissues, disturbances in enamel and dentin could have had a very wide spectrum of clinical manifestations [1]. The lesions may be localized only on one or two teeth or may be generalized with the changes seen in many teeth or in whole dentition. Moreover, the defects may be asymmetrical or symmetrical across the midline of the dentition [1]. In our research, five children had developmental defects of enamel in all teeth present in the oral cavity. In the case of three children, there was no information concerning the general disease since parents/caregivers did not return the questionnaire, one parent provided information that child had had cerebral palsy, and the other one only intellectual disability. However, this result suggests that the systemic factor or intoxicant operated for an extended period of enamel formation. General distribution might also be indicative of genetically determined amelogenesis imperfecta [5,6]. Although only one type of amelogenesis imperfecta (Kohlschütter-Tönz Syndrome) is associated with intellectual disability [9], there are some cases of patients with cerebral palsy affected by hypoplastic type of amelogenesis imperfecta described in the literature [31,32].

In the present research, the highest number of students (24.66%) with developmental defects of enamel was observed in the group of individuals with mild intellectual disability, whereas the lowest (11.11%) in those with profound disability. Interestingly, defects of early developing teeth appeared more frequently in subjects with moderate to profound forms of intellectual disability, which suggests that factors affecting the development of ectodermal structures during the prenatal and perinatal period and early childhood may lead to more serious neurological problems. However, interpretation of the detected between-group differences is difficult, due to underrepresentation of subjects with moderate to profound intellectual disability in the examined sample.

As far as dental caries indices are concerned, the results of the majority of studies are consistent in showing a positive relationship between caries and hypoplasia and/or demarcated enamel opacities and a negative association between diffuse fluoride-opacities and general amelogenesis imperfecta and dental caries [17,33,34,35]. Due to the screening character of the study, the prevalence of the particular types of enamel defects is unknown. However, more teeth with active caries were observed in students without enamel defects as compared to those with enamel defects. Similarly, the calculated DMFT index for students affected by enamel defects was lower (3.86 ± 2.69), while in those free of such changes, the index amounted to 5.03 ± 4.37. Thus, the defective enamel turned out to be resistant to caries, which is an unusual phenomenon that needs further investigation. One could argue that students with intellectual disability might have overdosed fluoride during childhood due to poor control of swallowing reflex and intensive dental caries prevention. There is also the possibility that some of them suffered from undiagnosed hereditary enamel defects. Nevertheless, numerous teeth in the examined population had caries and high DMFT was noted both in subjects with mild and with a more advanced degree of intellectual disability. Therefore, there is a need for intensive dental care, including prophylaxis and treatment, in these subjects. The present study also revealed that in special-care students from Poznan, enamel defects were observed less frequently in mandibular incisors (12.98%), whereas maxillary incisors were most often affected (49.52%), and then maxillary and mandibular premolars, 39.80% and 39.41%, respectively. The results are in agreement with other research studies concerning disabled as well as healthy individuals in which authors also found maxillary incisors as most frequently affected by developmental defects of enamel [2,12,14,36]. Interestingly, as suggested in the literature, the ameloblasts responsible for the production of thick enamel are more susceptible to systemic disorders in comparison to those that are associated with the thin enamel [12]. The process of calcium ion diffusion from the ameloblasts into the matrix of enamel, as well as the elimination of organic substances, are slower in the thick enamel in comparison to the thin tissue. Accordingly, the teeth are subject to systemic factors for a more extended period.

The prevalence of enamel defects in permanent teeth of special-care students from Poznan was lower than in the study of Modric et al. [3] who examined children with intellectual disability from Croatia (19.40% vs. 27.78%, respectively). However, results of both studies revealed that enamel defects were more often seen in maxillary teeth than in mandibular ones [3].

Singular defects, observed in five students, might have been caused by local factors such as traumatic injury to the primary tooth, compromising permanent successor development [37]. As emphasized in the literature, the upper central incisors are most often affected by traumatic injury [38,39,40]. In the study of Amorim et al. [37] the maxillary central incisors accounted for 83.3% of the teeth that sustained traumatic injuries, followed by maxillary lateral incisors (11.0%), mandibular central and lateral incisors (3.7%), canines and molars (2.0%). Traumatic injuries of the teeth and mouth may be observed during falls associated with loss of balance or epileptic seizures in patients with disability [38]. Moreover, in patients with disability, self-injury may occur within the maxillofacial area, oral tissues, and other areas of the body [41,42,43].

As suggested in the literature, intellectual disability may be another systemic factor related to the presence of developmental defects of enamel. The authors emphasized that early alterations in embryological development, hereditary agents, gestational or perinatal problems, external influences, and somatic disturbances during childhood are also etiologic factors of intellectual deficiency [2].

The study carried out among 470 children without disabilities (6–8 and 12–14 years old) from the Srem Commune in Wielkopolska province (Poland) revealed enamel defects within permanent incisors and first molars in 23.0% of the subjects (9.5% of molars and 10.1% of incisors showed developmental defects of enamel) [14]. The present research was performed in the same province, in the population exposed to similar fluoride content, yet it showed much higher prevalence rates (49.52% of maxillary incisors, 12.98% of mandibular incisors, and 17.35% of mandibular molars with enamel defects, respectively).

In the study by Martinez et al. [2], among 170 mentally disabled students aged 4–17 years old living in a non-fluoridated area, 37% had enamel defects of permanent teeth. Maxillary central incisors were most often affected (68.38%), and then mandibular central incisors (14.83%), maxillary premolars (10.96%) and mandibular premolars (5.16%). In our research, the distribution of the defects was similar, apart from mandibular incisors, which turned out to be the least frequently affected teeth (12.98%). Interestingly, as in Modric et al.’s [3] study, the upper central left incisor was the most commonly affected tooth. Finally, Jindal et al. [12] carried out the examination concerning developmental enamel lesions in 496 students, aged 8–15 years old, with various developmental disabilities (including intellectual disability, hearing impairment, locomotor handicaps, partial sight). They discovered that 40.90% of special needs children had developmental defects of enamel, whereas in the control group of healthy children the changes were observed in 5.40% of individuals.

It is difficult to reach a full comparison of our results with those of other authors due to the variability in age of the examined individuals and the number of erupted teeth. Moreover, there are often differences in the methodology used, such as additional cleaning and drying of teeth or type of lighting. The most comparable methodology was used during dental screenings financed from the Poznan City Council’s budget [44]. This study, involving 5634 5–15-year-old students from schools in the city of Poznan, revealed enamel defects of permanent dentition in 9.6% of examined children. Among them, 3.7% had diffuse opacities associated with low susceptibility to dental caries. Oral health examinations were carried out, in the same location, and with the use of similar methods as in the present study (dental screening in a school setting). They indicate that a relatively high percentage of enamel defects in this population might be resistant to caries, which could also explain the results of the present study concerning children with intellectual disability.

When it comes to gender, the literature data are in conflict as to whether girls or boys are more often affected by DDE [45,46]. Our present study failed to demonstrate any significant difference in the prevalence of DDE between boys and girls.

In addition, the present study has some other limitations that warrant discussion. Firstly, as was also noticed by Modric et al. [3], the optimal time for assessment of developmental defects of enamel is soon after the eruption of teeth, since there is a lack of stability of such findings because they could be lost by dental caries, trauma or attrition. However, to decrease this problem, we excluded from the study the individuals with a high number of teeth with severe carious lesions or those extracted. As a result, the mean age of participants with enamel defects and without lesions was similar (age did not affect the scope of our diagnosis). Secondly, not every special-care student with permanent dentition had oral examination done, because of a lack of parental consent. Therefore, the results could be different if all individuals had been involved. Thirdly, due to incomplete data from subjects’ medical history, the possible causation factors could not be determined. Needless to say, in the present research, DDE could be diagnosed on all surfaces except interdental surfaces. Therefore, it could be possible that DDE might be not diagnosed in those areas in the examined subjects. Furthermore, due to conditions of dental examination, i.e., out of dental surgery, with the use only of the light of the head lamp, enamel defects might be underestimated. On the other hand, some individuals did not have full permanent dentition because of unerupted second molars, as well as due to extractions. Thus, the students might have DDE in the lost teeth, which could change the final results. Finally, the researchers did not have data for individuals over the age of 20 since they had had to be excluded because the patients had extensive carious changes (5 subjects), or numerous extracted teeth (7 subjects), or the subjects were uncooperative during examination (45 subjects).

However, on the other hand, there are certain strengths in the present survey, since a large number of individuals from all special-care schools were examined, and all students participating in the research attended institutions localized in one large city. We could assume that they were living in the same environment, with exposition to similar environmental pollutants. Moreover, the study group was specifically selected since all participants had only permanent dentition. As a result of this, all teeth erupted and present in the oral cavity could be evaluated. We have not found any paper concerning the occurrence of enamel defects in such a homogenous and numerous group of subjects with intellectual disability. Therefore, presenting those results seems to be quite valuable, even if there is no information on personal risk factors concerning defects in enamel mineralization as well as the type of such changes.

This study provides characteristics of enamel defects as well as dental caries occurrence in subjects with intellectual disability attending all special-care schools in a large Polish city. Contrary to initial expectations, individuals with enamel defects did not present with higher dental caries indices which suggest that many of detected developmental abnormalities were caries-resistant. Thus, there is a need for further studies concerning the character and etiology of enamel defects in individuals with disability.

## 5. Conclusions 

The present study revealed that in special-care students from Poznan, developmental enamel defects and dental caries were frequently observed. However, contrary to initial expectations, individuals with developmental enamel defects did not show higher dental caries indices. There is a need for further studies concerning the etiology of enamel defects in the dentition of individuals with a disability.

## Figures and Tables

**Table 1:** Inclusion and exclusion criteria for the subjects in the study.

Inclusion Criteria	Exclusion Criteria
Parental written and informed consent	No parental written and informed consent
Child’s cooperativeness	Child’s uncooperativeness
Permanent dentition	Primary or mixed dentition
Caucasian origin	Other than Caucasian origin
Subjects from all eight special-care schools situated in Poznan	Subjects from special-care schools situated in other cities than Poznan
Subjects residing in Wielkopolska province, i.e., in cities neighboring to Poznan	Subjects from other provinces than Wielkopolska
Subjects present at school on days of examination	Subjects absent at school on days of examination
Group with the same ethnic, cultural, demographic, or regional origin	Other ethnic, cultural, demographic, or regional origin
The number of teeth extracted due to caries and/or teeth with crowns completely destroyed by decay < 6	Numerous teeth extracted and/or damaged by carious process in individual

**Table 2:** Number of special-care students with developmental defects of enamel (DDE) with respect to degree of their intellectual disability.

Degree of Intellectual Disability	Number of Examined Students		Number of Individuals with DDE		*p*	
	*N*	%	*N*	%		
Mild	146	100.00	36	24.66	A	A vs. B 0.03
Moderate	84	100.00	11	13.09	B	A vs. C ns
Severe	29	100.00	4	13.79	C	A vs. D ns
Profound	9	100.00	1	11.11	D	B vs. C ns
Moderate to profound	122	100.00	16	13.11	E	A vs. E 0.02
Total	268	100.00	52	19.40		B vs. D ns C vs. D ns

N—number of subjects; DDE—developmental defects of enamel; A—percentage of patients with DDE in a group with mild intellectual disability; B—percentage of patients with DDE in a group with moderate intellectual disability; C—percentage of patients with DDE in a group with severe intellectual disability; D—percentage of patients with DDE in a group with profound intellectual disability; E—percentage of patients with DDE in a group with moderate to profound intellectual disability; ns—not significant: *p* > 0.05.

**Table 3:** Percentage of females and males affected by DDE, and age of students with DDE and without DDE.

	Students with DDE	Students without DDE
Sex		
Females *n* (%)	25 (21.20%)	93 (78.80%)
Males *n* (%) *	27 (18.00%)	123 (82.00%)
Age (years)		
Range	10–19	10–20
Median	14.00	15.00
Mean	14.67 **	14.80
SD	1.85	2.21

SD—standard deviation; ns—not significant: *p* > 0.05; * ns (*p* = 0.5126) as compared to percentage of females affected by DDE; ** ns (*p* = 0.7292) as compared to the age of students without DDE.

**Table 4:** Number of special-care students with DDE of early and late developing teeth with respect to degree of their intellectual disability.

Degree of Intellectual Disability	Number of Individuals with DDE of First Permanent Molars and Incisors		Number of Individuals with DDE Limited to Canines, Premolars and Second Molars		*N*		*P*
	*N*	%	*N*	%			
Mild	23	60.53	13	39.47	A	36	A vs. B 0.02
Moderate to profound	15	93.75	1	6.25	B	16	
Total	38	73.08	14	19.40		52	

ns—not significant: *p* > 0.05; A—percentage of patients with defects if early developing teeth in a group with mild intellectual disability; B—percentage of patients with defects of early developing teeth in a group with moderate to profound intellectual disability.

**Table 5:** Dental caries intensity expressed as DMFT number with respect to degree of intellectual disability.

Degree of Intellectual Disability	DMFT					*p*
	Mean ± SD	Median	*N* (%)	Range		
Mild	4.56 ± 3.85	4.00	146 (54.48)	0–28	A	A vs. B ns
Moderate to profound	5.09 ± 4.42	4.00	122 (45.52)	0–28	B	
Total	4.80 ± 4.12	4.00	268 (100.00)	0–28		

ns—not significant: *p* > 0.05; *N*—number of subjects; % percentage of subjects; A—DMFT of patients with mild intellectual disability; B—DMFT of patients with moderate to profound intellectual disability.

**Table 6:** Number of teeth with DDE in special-care students.

Number of Teeth with DDE	Number of students			*p*	
	*N*	%			
1	8	15.39	A	A vs. B	ns
2	11	21.15	B	A vs. C	ns
3	3	5.78	C	A vs. D	ns
4	7	13.46	D	A vs. E	0.03
5	3	5.78	C	A vs. F	0.008
6	2	3.85	E	A vs. G	ns
8	2	3.85	E	B vs. C	0.02
10	2	3.85	E	B vs. D	ns
12	1	1.92	F	B vs. E	0.005
13	1	1.92	F	B vs. F	0.002
14	1	1.92	F	B vs. G	ns
16	2	3.85	E	C vs. D	ns
21	1	1.92	F	C vs. E	ns
22	1	1.92	F	C vs. F	ns
23	1	1.92	F	C vs. G	ns
24	1	1.92	F	D vs. E	ns
27	1	1.92	F	D vs. F	0.02
28	4	7.69	G	D vs. G	ns
Total	52	100.00		E vs. F	ns
				E vs. G	ns
				F vs. G	ns

ns–not significant: *p* > 0.05.

**Table 7:** Number of teeth groups present in the oral cavity and those affected by enamel opacities with respect to the dental arch (maxillary and mandibular) as well as to the side of the oral cavity (right and left).

Groups of Teeth		Maxillary (Mx) Teeth		Mandibular (Mb) Teeth		*p* Maxillary vs. Mandibular Teeth	Right Teeth		Left Teeth		P Right vs. Left Teeth	Total			*p*	
		*N*	%	*N*	%		*N*	%	*N*	%		*N*	%			
Incisors	Total number of teeth	208	100.00	208	100.00	-	208	100.00	208	100.00	-	416	100.00			
	Teeth with opacities	103	49.52	27	12.98	<0.001	61	29.33	69	33.17	ns	130	31.25	A	A vs. B	ns
Canines	Total number of teeth	103	100.00	104	100.00	-	104	100.00	103	100.00	-	207	100.00		A vs. C	0.02
	Teeth with opacities	39	37.86	24	23.08	0.02	32	30.77	31	30.10	ns	63	30.43	B	A vs. D	<0.001
Premolars	Total number of teeth	206	100.00	203	100.00	-	206	100.00	203	100.00	-	409	100.00		B vs. C	0.03
	Teeth with opacities	82	39.80	80	39.41	ns	83	40.29	79	38.92	ns	162	39.61	C	B vs. D	<0.001
Molars	Total number of teeth	206	100.00	207	100.00	-	205	100.00	208	100.00	-	413	100.00		C vs. D	<0.001
	Teeth with opacities	41	19.90	34	17.35	ns	39	19.02	36	17.31	ns	75	18.16	D		
Total	Total number of teeth	723	100.00	722	100.00	-	723	100.00	722	100.00	-	1445	100.00			
	Teeth with opacities	265	36.65	165	22.85	<0.001	215	29.74	215	29.78	ns	430	29.76			

ns—not significant: *p* > 0.05; A—percentage of incisors with DDE; B—percentage of canines with DDE; C—percentage of premolars with DDE; D—percentage of molars with DDE.

**Table 8:** Number of students with enamel opacities with respect to particular groups of teeth (at least one tooth had to be affected in the group).

Groups of Teeth				Number of Patients			*p*	
Incisors	Canines	Premolars	Molars	*N*	%			
+	-	−	−	19	36.54	A	A vs. B	<0.001
-	+	−	−	2	3.85	B	A vs. C	0.006
-	-	+	−	7	13.46	C	A vs. D	<0.001
-	-	−	+	0	0.00		A vs. E	<0.001
+	+	−	−	2	3.85	B	A vs. F	ns
+	-	+	-	2	3.85	B	B vs. C	ns
+	-	−	+	2	3.85	B	B vs. D	ns
-	+	+	−	3	5.77	D	B vs. E	ns
-	+	−	+	1	1.92	E	B vs. F	0.01
-	-	+	+	1	1.92	E	C vs. D	ns
+	+	+	−	3	5.77	D	C vs. E	0.03
+	+	−	+	0	0.00		C vs. F	ns
+	+	+	+	10	19.23	F	D vs. D	ns
Total				52	100.00		D vs. F	0.04
							E vs. F	0.04

“+” – group of teeth affected by DDE; “-“ – group of teeth without DDE; ns—not significant: *p* > 0.05.

**Table 9:** Comparison of the number of teeth groups affected by caries (with carious cavities, fillings and extracted due to decay) between subjects with and without enamel opacities.

Group of Teeth	Subjects with DDE					Subjects without DDE					*p*		Total			Total			All Subjects		
	Maxillary (Mx) Teeth		Mandibular (Mdb) Teeth		*p* Mx vs. Mdb Teeth	Maxillary (Mx) Teeth		Mandibular (Mdb) Teeth		*p* Mx vs. Mdb Teeth	For Total		Subjects with DDE			Subjects without DDE			In Total		
	N	%	N	%		N	%	N	%				N	%	*p*	N	%	*p*	N	%	*p*
Incisors	4	1.92	5	2.40	ns	99	11.56	40	4.67	<0.001	A	A vs. B A vs. C	9	2.16	0.03 <0.001	139	8.12	<0.001 0.04	148	6.95	<0.001 <0.001
Canines	0	0.00	0	0.00	ns	8	1.92	6	1.41	Ns	B	A vs. D B vs. C	0	0.00	<0.001 <0.001	14	1.66	<0.001 <0.001	14	1.33	<0.001 <0.001
Premolars	19	9.22	13	6.40	ns	93	11.10	83	9.90	Ns	C	B vs. D C vs. D	32	7.82	<0.001 <0.001	176	10.50	<0.001 <0.001	208	9.98	<0.001 <0.001
Molars	57	27.67	103	49.76	<0.001	305	38.22	451	54.73	<0.001	D		160	38.74		756	46.61		916	45.01	
Total	80	11.06	121	16.76	0.005	505	17.36	580	19.71	0.003			201	13.91		1085	18.54		1286	17.62	

Mx—maxillary; Mdb—mandibular; ns—not significant: *p* > 0.05.
